# Association between neuroserpin and molecular markers of brain damage in patients with acute ischemic stroke

**DOI:** 10.1186/1479-5876-9-58

**Published:** 2011-05-11

**Authors:** Raquel Rodríguez-González, Tomás Sobrino, Manuel Rodríguez-Yáñez, Mónica Millán, David Brea, Elena Miranda, Octavio Moldes, Juan Pérez, David A Lomas, Rogelio Leira, Antoni Dávalos, José Castillo

**Affiliations:** 1 Clinical Neuroscience Research Laboratory, Department of Neurology, Hospital Clínico Universitario, University of Santiago de Compostela, Santiago de Compostela, Spain; 2 Department of Neurosciences, Hospital Germans Trias i Pujol, Universitat Autònoma de Barcelona, Spain; 3 University of Cambridge, Cambridge Institute for Medical Research, Cambridge, UK; 4 Departamento de Biología Celular, Genética y Fisiología, Universidad de Málaga, Facultad de Ciencias, Campus de Teatinos, Málaga, Spain

## Abstract

**Background:**

Neuroserpin has shown neuroprotective effects in animal models of cerebral ischemia and has been associated with functional outcome after ischemic stroke. Our aim was to study whether neuroserpin serum levels could be associated to biomarkers of excitotoxicity, inflammation and blood brain barrier disruption.

**Methods:**

We prospectively included 129 patients with ischemic stroke (58.1% male; mean age, 72.4 ± 9.6 years) not treated with tPA within 12 hours (h) of symptoms onset (mean time, 4.7 ± 2.1 h). Poor functional outcome at 3 months was considered as a modified Rankin scale score >2. Serum levels of neuroserpin, Interleukin 6 (IL-6), Intercellular adhesion molecule-1 (ICAM-1), active Matrix metalloproteinase 9 (MMP-9), and cellular fibronectin (cFn) (determined by ELISA) and glutamate (determined by HPLC) were measured on admission, 24 and 72 h. The main variable was considered the decrease of neuroserpin levels within the first 24 h. ROC analysis was used to select the best predictive value for neuroserpin to predict poor functional outcome due to a lack of linearity.

**Results:**

The decrease of neuroserpin levels within the first 24 h was negatively correlated with serum levels at 24 hours of glutamate (r = -0.642), IL-6 (r = -0.678), ICAM-1 (r = -0.345), MMP-9 (r = -0.554) and cFn (r = -0.703) (all P < 0.0001). In the multivariate analysis, serum levels of glutamate (OR, 1.04; CI95%, 1.01-1.06, p = 0.001); IL-6 (OR, 1.4; CI95%, 1.1-1.7, p = 0.001); and cFn (OR, 1.3; CI95%, 1.1-1.6, p = 0.002) were independently associated with a decrease of neuroserpin levels <70 ng/mL at 24 h after adjusting for confounding factors.

**Conclusions:**

These findings suggest that neuroprotective properties of neuroserpin may be related to the inhibition of excitotoxicity, inflammation, as well as blood brain barrier disruption that occur after acute ischemic stroke.

## Background

Several studies have shown that the serin protease inhibitor, neuroserpin, exerts a neuroprotective effect after brain ischemia, probably due to its natural ability to form an inactivating complex with tissue plasminogen activator (tPA). It is also known that tPA is able to promote neuronal injury in the brain parenchyma by enhancing different mechanisms, such as the activation of microglia [1] as well as affecting neuronal N-methyl-D-aspartate (NMDA) receptor-mediated signalling [2]. This leads to an increased release of cytotoxic agents, such as inflammatory mediators, a matrix metalloproteinase-mediated digestion of the extracellular matrix and a glutamate-induced excitotoxicity. The effect of neuroserpin on reducing this tPA-induced damage in the brain has been studied, and both the overexpression of neuroserpin [3] and neuroserpin treatment after cerebral ischemia [4,5] have proved to be effective in reducing the final lesion.

Furthermore, an association between neuroserpin serum levels and functional outcome in patients with ischemic stroke has recently been reported [6]. In the present study, we sought to investigate whether neuroserpin serum levels in patients with ischemic stroke could be associated to serum levels of different molecules of the ischemic cascade. Thus, glutamate was measured as a marker of excitotoxic damage, interleukin-6 (IL-6) and Intercellular Adhesion Molecule-1 (ICAM-1) as markers of inflammatory response, and matrix metalloproteinase 9 (MMP-9) and cellular fibronectin (cFn) as markers of blood brain barrier disruption after ischemic stroke.

## Patients and methods

### Study population and patients characteristics

One hundred and ninety patients with a first-ever ischemic stroke of less than 12 hours from symptoms onset, and previously independent for their daily living activities, were prospectively evaluated to be included in the study. Patients with chronic inflammatory diseases (n = 5), severe hepatic (n = 4), renal (n = 2) or hematological diseases (n = 2), cancer (n = 4) or infectious disease in the 15 days prior to inclusion (n = 5) were excluded due to their impact on stroke outcome and possible interference in neuroserpin levels. Sample size was calculated using EPIDAT software http://www.sergas.es/MostrarContidos_N3_T01.aspx?IdPaxina=62715 assuming alpha and beta errors of 0.05 and 0.2, respectively.

Likewise, 26 patients who had received thrombolytic treatment were excluded in order to assess the neuroprotective role of neuroserpin without the disturbance of rtPA. Seven patients did not accept to participate and 6 patients were lost during the follow-up, therefore, a total of 129 patients were finally included in the study. This research was carried out in accordance with the Declaration of Helsinki of the World Medical Association (2000) and approved by the Ethics Committee of the participating hospital. Informed consent was obtained from each patient or their relatives after full explanation of the procedures.

### Clinical variables

All patients were admitted to an acute stroke unit and treated following the European Stroke Organization guidelines [7]. Medical history recording potential vascular risk factors, blood and coagulation tests, 12-lead ECG, chest radiography, and carotid ultrasonography were performed on admission. Stroke subtype was classified according to the TOAST criteria as atherothrombotic (n = 23), cardioembolic (n = 54), lacunar (n = 15), and undetermined (n = 37) [8]. Stroke severity was assessed by a internationally certified neurologist using the National Institute of Health Stroke Scale (NIHSS) on admission, 24 ± 6 hours, 48 ± 6 hours, 72 ± 24 hours, and at 7 ± 1 and 90 ± 7 days. Early neurological deterioration (END) was diagnosed in those patients who worsened 4 or more points on NIHSS score within the first 48 hours. Functional outcome was evaluated at 3 months using the modified Rankin Scale (mRS), considering a score >2 as poor outcome.

### Neuroimaging variables

CT scans were carried out on admission and between days 4 and 7. Infarct volume was calculated in the second CT by using the formula 0.5 × a × b × c, where a and b are the largest perpendicular diameters, and c is the number of 1-cm thick sections that contain the lesion. All neuroimaging evaluations were made by the same neuroradiologist who had no knowledge of the patients' clinical and laboratory results.

### Laboratory determinations

Serum glucose, platelet count and coagulation tests were assessed in a central laboratory. Blood samples, drawn from all patients on admission, at 24 ± 6 and 72 ± 24 hours, were collected in glass chemistry test tubes, centrifuged at 3000 xg for 10 minutes, and serum immediately frozen and stored at -80°C until analysis. Glutamate levels, as a biomarker of excitotoxicity, were determined by HPLC, using the Waters Pico Tag^® ^Chemistry Package for HPLC amino acids analysis. IL-6 and ICAM-1, as indexes of inflammatory response, were determined by IMMULITE 1000 System (Siemens) and a commercially available sandwich enzyme-linked inmunosorbent assay (ELISA) kit from Bender Medsystems, respectively. Finally, as blood brain barrier disruption markers, active MMP-9 and cFn were determined using GE Healthcare and Biohit Plc ELISA kits, respectively. For neuroserpin quantification, a sandwich ELISA was performed as described previously [6,9,10]. Each sample was assayed in duplicate and intra-assay coefficients of variation sample values were always <15%. Clinical investigators were unaware of the laboratory results until the end of the study, once the database was closed. The absolute difference between basal and 24 hours neuroserpin levels was defined as neuroserpin decrease. All determinations were carried out in a laboratory blind to the clinical outcome and neuroimaging findings.

### Endpoints

The primary endpoint was the decrease of neuroserpin levels at 24 hours.

### Statistical analysis

For continuous variables, we tested if data presented a Normal distribution using the Kolgomorov-Smirnov test. Parametric tests were used if they followed a Normal distribution and non-parametric tests if they did not. Mann-Whitney test was used for continuous variables with non-Normal distribution, Student's t test for continuous variables with Normal distribution and Chi-square test for proportions between patients. In addition Spearman analysis was used for bivariate correlations with non-Normal distribution. Results are expressed as percentages for categorical variables and as mean (SD) or median [quartiles] for the continuous variables depending on their normal distribution or not. Neuroserpin was used as a continuous variable since there was a linearity of the odds ratios for outcome. The influence of neuroserpin decrease at 24 h on serum levels of molecular markers of brain injury was assessed by logistic regression analysis, after adjusting for the main baseline variables related to neuroserpin decrease in the univariate analysis (enter approach and probability of entry P < 0.05). Due to a lack of linearity, the decrease of neuroserpin levels at 24 hours was categorized by ROC analysis. Results were expressed as adjusted odds ratios (OR) with the corresponding 95% confidence intervals (95% CI). The statistical analysis was conducted using SPSS 16.0 for Windows XP.

## Results

A total of 129 patients (58.1% male; mean age 72.4 ± 9.6 years) who did not receive thrombolytic treatment were prospectively included in the study within 12 hours of symptoms onset (mean time 4.7 ± 2.1 hours). The NIHSS score on admission was 9 [4,14] . Neuroserpin levels on admission were significantly greater [148.4 ± 37.7 ng/mL] than at 24 hours [79.1 ± 52.3 ng/mL] or at 72 hours [80.9 ± 60.5 ng/mL] (all p <0.0001). No differences in neuroserpin levels between 24 and 72 hours were found. Median neuroserpin decrease within the first 24 hours was 69.4 ± 51.5 ng/mL.

A decrease in neuroserpin levels <70 ng/mL within the first 24 hours predicted the probability of poor outcome (area under curve 0.921, P < 0.0001) with the highest sensitivity (84%) and specificity (91%). For this reason, our analysis focused on those molecular markers of brain injury which were positively associated with a decrease in neuroserpin levels <70 ng/mL at 24 hours.

### Neuroserpin and brain injury biomarkers

**Figure 1:**
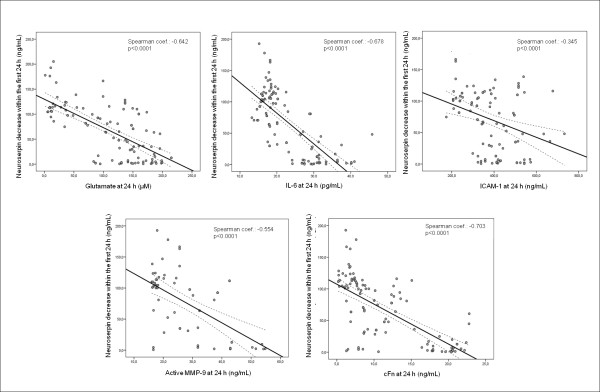
**Significant correlations between neuroserpin decrease within the first 24 hours and levels of molecular markers of brain damage at 24 hours**.

We evaluated the relationship between neuroserpin levels and brain injury biomarkers on admission as well as between neuroserpin decrease within the first 24 hours and biomarkers serum levels at 24 hours from stroke onset. We did not find a significant correlation between neuroserpin serum levels on admission and glutamate (r = -0.138, p = 0.133), IL-6 (r = -0.062, p = 0.485), ICAM-1 (r = 0.004, p = 0.964), active MMP-9 (r = 0.143, p = 0.224) or cFn (r = -0.139, p = 0.117). However, the decrease of neuroserpin levels within the first 24 h was negatively correlated with serum levels of brain injury biomarkers at 24 hours: glutamate (r = -0.642), IL-6 (r = -0.678), ICAM-1 (r = -0.345), active MMP-9 (r = -0.554), cFn (r = -0.703), (all P < 0.0001) (Figure 1).

Our analysis showed that patients with a decrease of neuroserpin levels within the first 24 hours < 70 ng/mL presented greater serum levels of glutamate, IL-6, ICAM-1, active MMP-9 and cFn at 24 hours (Table 1). In the multivariate analysis, serum levels of glutamate (OR, 1.04; CI 95%, 1.01-1.06, p = 0.001), IL-6 (OR, 1.4; CI 95%, 1.1 - 1.7, p = 0.001) and cFn (OR, 1.3; CI 95%, 1.1 - 1.6, p = 0.002) were independently associated with a decrease of neuroserpin levels < 70 ng/mL after adjustment for age, sex, previous stroke, lesion volume, glucose levels and baseline stroke severity (Table 2).

**Table 1:** Univariate analysis for neuroserpin decrease.

	Neuroserpin decrease within first 24 h ≥70 ng/mL n = 70	Neuroserpin decrease within first 24 h <70 ng/mL n = 59	p
Female, %	30.0	55.9	0.003
Age, years	69.9 ± 10.6	75.2 ± 7.5	0.004
Time from onset, h	4.8 ± 2.2	4.5 ± 2.1	0.816
TOAST			0.051
- Atherothrombotic, %	17.1	18.6	
- Cardioembolic, %	35.7	49.2	
- Lacunar, %	18.6	3.4	
- Indeterminated, %	28.6	28.8	
History of hypertension, %	57.1	69.5	0.103
History of diabetes, %	22.9	28.8	0.284
History of dyslipemia, %	21.4	25.4	0.371
History of atrial fibrillation, %	20.0	32.2	0.084
Previous stroke, %	2.9	15.3	0.013
Systolic BP on admission, mm Hg	147.9 ± 23.6	145.6 ± 18.8	0.885
Diastolic BP on admission, mm Hg	81.3 ± 14.2	71.6 ± 12.2	0.062
Maximum temperature 24 h (°C)	36.7 ± 0.4	36.7 ± 0.5	0.943
Glycemia, mg/dL	120.4 ± 25.3	183.7 ± 87.4	0.001
Leukocyte count, 10^3^/mL	8.7 ± 2.4	9.2 ± 2.8	0.427
Fibrinogen, mg/dL	386.9 ± 105.2	428.1 ± 136.2	0.080
Early neurological deterioration, %	2.9	25.4	<0.0001
NIHSS on admission	5 [3,10]	14 [10,16]	<0.0001
Infarct volume, mL	18.2 ± 20.9	44.4 ± 36.3	<0.0001
**Molecular markers of brain damage**			
Glutamate 24 h, μM	67.7 ± 54.4	149.9 ± 36.3	<0.0001
IL-6 24 h, pg/mL	18.4 ± 3.2	29.2 ± 8.1	<0.0001
ICAM-1 24 h, ng/mL	344.7 ± 122.5	430.2 ± 79.1	<0.0001
Active MMP-9 24 h, ng/mL	23.9 ± 8.7	32.3 ± 11.1	<0.0001
cFn 24 h, μg/mL	7.6 ± 1.9	13.1 ± 5.6	<0.0001

Baseline clinical characteristics, stroke subtype, vascular risk factors, biochemical parameters, neuroimaging findings and molecular markers of brain damage in patients with a neuroserpin decrease within the first 24 hours ≥ 70 ng/mL or < 70 ng/mL.

**Table 2:** Adjusted OR of neuroserpin decrease levels <70 ng/mL at 24 hours for serum levels of glutamate, IL-6, ICAM-1, active MMP-9 and cFn at 24 hours.

	Adjusted OR (95% CI)	p
Glutamate at 24 hours	1.04 (1.01 to 1.06)	0.001
IL-6 at 24 hours	1.4 (1.1 to 1.7)	0.001
ICAM-1 at 24 hours	1.0 (0.9 to 1.1)	0.065
Active MMP-9 at 24 hours	1.1 (0.9 to 1.3)	0.095
cFn at 24 hours	1.3 (1.1 to 1.6)	0.002

Adjusted for sex, age, previous stroke, glucose levels, NIHSS on admission and infarct volume.

## Discussion

Neuroserpin has extensively shown neuroprotective activity after brain ischemia in experimental models [3-5]. In addition, an association between neuroserpin levels and acute ischemic stroke outcome has recently been reported [6]. However, the mechanisms that are involved in neuroserpin-mediated neuroprotection remain to be well characterized. In order to investigate this, the present study has explored the association between neuroserpin serum levels and established biomarkers of different mechanisms of brain injury which take place after acute ischemic stroke.

The implication of the selected biomarkers in different pathophysiological mechanisms that are triggered by ischemic stroke as well as their clinical value, have been extensively investigated and validated in previous studies carried out by our group as well as by others [10-20]. Hence, glutamate was selected as a biomarker of excitotoxic damage, ICAM-1 and IL-6 as inflammatory biomarkers, and MMP-9 as well as cFn as blood brain barrier disruption biomarkers. We did not find any significant statistical relationship between serum levels of neuroserpin and the selected biomarkers at baseline. However, a negative correlation was found between serum levels of all the biomarkers at 24 hours and neuroserpin decrease within the first 24 hours after stroke onset. Using ROC analysis, we had established a 70 ng/mL cut-off value for the decrease of neuroserpin serum levels within the first 24 hours to predict poor outcome. The results of the present study show a significant association between neuroserpin decrease < 70 ng/mL and serum levels of brain injury biomarkers at 24 hours, which remained independent for glutamate, IL-6 and cFn after adjusting for confounding factors.

Neuroserpin displays a neuroprotective effect in rodent models of cerebral ischemia [3-5] by inhibiting extravascular deleterious effects of tPA in the brain parenchyma. Due to the fact that patients treated with tPA were excluded in our study, neuroserpin would presumably be acting on endogenous tPA, whose expression increases after brain ischemia [4,21].

It has been demonstrated that tPA exacerbates glutamate-mediated excitotoxicity by its interaction with NMDA receptor [22,23] and also that neuroserpin is able to protect neurons from NMDA-induced neuronal death both in vitro and in vivo [24], probably by limiting this deleterious tPA-mediated effect on glutamatergic signalling. Our results show a significant relationship between a greater neuroserpin decrease in serum within the first 24 h after stroke onset and lower glutamate serum levels at 24 hours. This result seems to be in accordance with experimental studies, suggesting that neuroserpin might affect glutamate-mediated excitotoxic response after ischemic stroke.

We have also found significant relationships between a greater neuroserpin decrease within the first 24 hours from stroke onset and lower levels of the inflammatory biomarkers ICAM-1 and IL-6 at 24 hours. Previous studies by our group have reported associations between serum levels of these inflammatory markers and clinical features such as early neurological deterioration, greater final infarct volume and cerebral edema [14,25]. It is known that tPA, whose expression increases after brain ischemia, activates microglial cells which produce inflammatory molecules that promote neuronal damage [26,27]. In addition, some of these molecules, like tumour necrosis factor alpha (TNF-α) or interleukin-1 beta (IL-1β) strongly up-regulate the expression of adhesion molecules such as ICAM-1 [28], thus contributing to the extension of the lesion. Likewise, the extracellular matrix substrate fibronectin is able to promote microglial activation [29-31].

It has also been proposed that neuroserpin could reduce microglial activation after ischemic stroke due to its ability to form tPA-inactivating complexes in the brain parenchyma [3]. Because neuroserpin serum levels within the first 24 hours are associated with a lower level of the inflammatory biomarkers ICAM-1 and IL-6 at 24 hours, we hypothesize that those patients who show more severe clinical outcome might require more neuroserpin in the brain parenchyma to inactivate tPA, and this could lead to lower neuroserpin levels in serum. Neuroserpin, via complex formation with tPA, could limitate microglial activation, therefore the production of inflammatory mediators would be reduced, as reflected by the decreased serum levels observed.

Likewise, after ischemia, an increase in neuroserpin contributes to preserving the integrity of the basement membrane [4] and decreases blood brain barrier leakage, reducing ischemic lesion [5]. MMP-9 is an endopeptidase which mediates extracellular matrix degradation, and glycoprotein fibronectin is one of its substrates [32]. There is abundant evidence indicating that increased MMP-9 expression after ischemia significantly contributes to basal lamina degradation, thus leading to hemorrhagic transformation of ischemic stroke [18,33-36]. It has also been proved that tPA enhances MMP-9 expression in vitro and in vivo [37,38]. Furthermore, tPA-treated patients show increased plasma levels of MMP-9 [39]. Recent results from our group have also shown a negative correlation between neuroserpin decrease within the first 24 hours and MMP-9 levels at 24 hours in patients treated with tPA [6], which is in line with the results of the present manuscript, where a greater decrease in neuroserpin serum levels within the first 24 hours was correlated with lower serum levels of MMP-9 at 24 hours. Accordingly, we postulate that greater expression of neuroserpin in the brain parenchyma could contribute to stronger downregulation of tPA activity, therefore, reducing tPA-induced MMP-9 expression.

## Conclusions

In conclusion, we have found a negative correlation between the decrease in neuroserpin serum levels within the first 24 hours and levels of molecular markers of brain damage at 24 hours after ischemic stroke. We suggest that neuroprotective properties of neuroserpin might be related to the inhibition of tPA-mediated mechanisms of excitotoxicity, inflammation, as well as blood brain barrier disruption that occur after acute ischemic stroke. This is in line with recent results from our group obtained after investigating neuroserpin effects using an in vitro model of brain ischemia [40].

The information reported here regarding biomarkers might be relevant to evaluate the utility of neuroserpin as a potential treatment for ischemic stroke patients. In this respect, combined thrombolytic and neuroprotective therapy continues to be one of the most interesting approaches for ischemic stroke. This and future studies could contribute to better molecular characterization of the deleterious consequences of thrombolytic therapy, and lead to the development of effective strategies to reduce them.

## Competing interests

The authors declare that they have no competing interests.

## Authors' contributions

RRG, TS, RL, AD, JC have conceived and designed the research; analyzed and interpreted the data; performed statistical analysis, handled funding and supervision and drafted the manuscript. RRG, DB, OM, have acquired, analyzed and interpreted the molecular data, and made supervision. EM, JP, DAL, have provided the materials and technical advice with the development of the neuroserpin ELISA used in the study. MRY, MM, helped to acquired, analyzed and interpreted the clinical data and made critical revision of the manuscript. All authors read and approved the final manuscript.
